# Applicability of Fractal Analysis for Quantitative Evaluation of Midpalatal Suture Maturation

**DOI:** 10.3390/jcm12134189

**Published:** 2023-06-21

**Authors:** Ali Farid Darawsheh, Béla Kolarovszki, Da Hye Hong, Nelli Farkas, Soroush Taheri, Dorottya Frank

**Affiliations:** 1 Department of Dentistry, Oral and Maxillofacial Surgery, Medical School, University of Pécs, 7624 Pécs, Hungary; 2 Institute of Bioanalysis, Medical School, University of Pécs, 7624 Pécs, Hungary

**Keywords:** suture, fractal, cone beam computed tomography, maxillary, constriction

## Abstract

Background: The treatment of transversal maxillary deficiency usually aims at skeletal expansion. The treatment option highly depends on the maturation stage of the midpalatal suture (MPS), which may vary between individuals at the same chronological age. Therefore, the individual determination of the MPS maturation is crucial. Aims: Our aim was to investigate the applicability of fractal analysis for the quantitative evaluation of MPS maturation. Methods: Nine experienced orthodontists were asked to evaluate the MPS maturation on 51 cone beam computed tomography (CBCT) scans according to the Angelieri classification method. Intra- and inter-examiner reliability was measured using Cohen’s Kappa coefficient. The stages were agreed upon according to the results of the examiners with the highest strength of agreement. Fractal analysis was then performed on the CBCTs and the correlation between the fractal dimension values and maturation stages was then evaluated. Optimal fractal dimension cut-off values were determined using a receiver operating characteristic curve. Results: The cut-off point was found at 1.073, at which the use of fractal dimension for predicting MPS maturation showed 100% sensitivity, 93.7% specificity, 9.5% false positive, 0% false negative rate. Conclusion: Our results provided further evidence that fractal analysis is a reliable tool to determine MPS maturation stage.

## 1. Introduction

In orthodontics, several treatment possibilities exist for the correction of transversal maxillary deficiency, with the overall objective of splitting the midpalatal suture (MPS) and circummaxillary suture systems to widen the maxilla [1]. Among the available options, rapid maxillary expansion (RME) is the most widely used technique used by clinicians for opened MPS [2]. The separation of MPS leads to a true increase in the transversal width via the formation of new bone at the site, hence correcting the transversal maxillary constriction [3]. By using RME, we can not only solve transversal discrepancies but relive dental crowding, improve function and smile esthetics, and further facilitate the correction of sagittal (Class II and Class III) problems as well [4,5,6].

Successful RME therapy depends on the current stage of craniofacial development and the prediction of the patient’s future growth potential [7]. Although maturation stage is a major determinant for treatment options (possible RME or more invasive approaches such as surgically assisted expansion, bone anchored maxillary expansion, mini-screw assisted maxillary expansion, transpalatal distraction), a great variability in the time of MPS maturation regarding the start and progression of fusion has been reported [8]. Namely, some authors have found the fusion to occur between 15 and 19 years of age; however, in some rare cases, even elderly people with opened sutures have been found [9,10,11]. Previous studies have found that 54- and 71-year-old individuals were exceptions to the early fusion rule [9,10]. According to these, the chronological age is not a justifiable indicator to determine the developmental status of the MPS [8].

The ability to predict the maturation stage of the MPS would greatly aid the proper therapeutic decision with the most optimal result. In recent years, various approaches have been used to obtain knowledge regarding the MPS, including the morphology, development, ossification, and suture closure [8,12,13]. In 2013, Angelieri et al. presented a novel classification method to individually assess the MPS maturation through cone beam computed tomography (CBCT) images of the suture [8]. The major clinical application of the MPS maturation classification is to classify patients into different MPS maturational stages and decide whether the patient is a suitable candidate for conventional RME or not [14].

In a previous study, Angelieri’s method was found to be potentially reliable and reproducible; however, the results of the study did not support that statement as the overall inter-examiner agreement ranged between 0.34 and 0.37, while the intra-examiner agreement ranged between 0.41 and 0.44 on the first and second test, respectively [7]. Therefore, it should be used with caution for routine applications. In 2016, Kwak et al. investigated the potential use of fractal analysis, a quantitative method for MPS maturation evaluation, and found a strong negative correlation between fractal dimension and MPS maturation [13].

Without doubt, an optimal diagnostic test must be reliable, reproducible, and practical [15]. A method can be applied in clinical settings if it is valid and reliable [16]. Currently, there is no reliable diagnostic tool available for the precise estimation of the MPS maturation stage which could highly contribute to a successful treatment outcome of transversal maxillary discrepancies. Therefore, the purpose of this present study was to test the applicability of fractal analysis for the quantitative evaluation of MPS maturation using CBCT to provide further aid for orthodontic diagnosis and therapeutic decisions.

## 2. Materials and Methods

### 2.1. CBCT Scans

The study was approved by the Regional Committee for Research Ethics (9107-PTE2022.). In total, 51 CBCT scans of untreated subjects with complete visualization of the MPS (19 men, 32 women, average age: 22.45) were randomly selected at the Department of Dentistry, Oral and Maxillofacial Surgery, University of Pécs. The obtained images were taken under the same voltage, with high resolution, complete visualization of different maturation stages of MPS, and free from pathological lesions. The detailed settings for the CBCT device were as follows: CBCT scan (GENDEX DP-800, Gendex, Hatfield, PA, USA), 90 kV and 5 mA, FOV: 8 × 15 cm, acquisition time 8.1 s, 775 mGy/cm^2^. Table 1 summarizes the age and gender distribution of the subjects.

The head positions on all the images were first adjusted according to the recommendation of Angelieri et al. [8] to standardize and allow consistent assessments of the MPS. 

### 2.2. Fractal Analysis

Fractal analysis was performed according to the method described previously [13]. Briefly, the previously evaluated CBCT scans were cropped close to the MPS, distal to the incisive canal and extending to the front of the posterior nasal spine. Following that, images were further edited. ImageJ software (National Institutes of Health and the Laboratory for Optical and Computational Instrumentation, LOCI, University of Wisconsin, Madison, WI, USA) was used for binary transformation and to reduce further noise by erosion and dilation. The images were then further skeletonized, and the box-counting function of the software was used to determine fractal dimension. 

### 2.3. Assessment of MPS Maturation Stages According to Angelieri’s Classification Method

**Figure 1:**
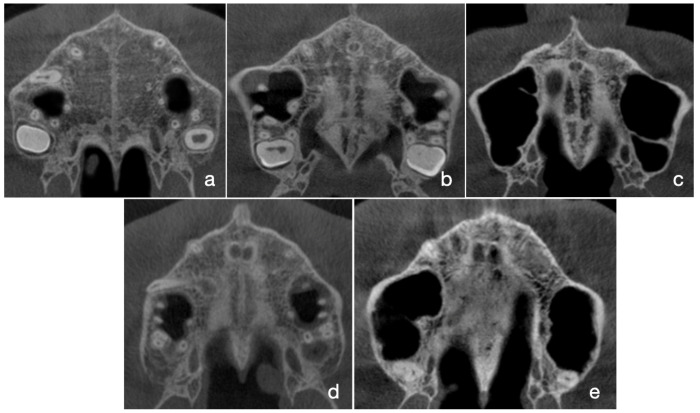
Representative CBCT images of the five maturation stages. At stage A, only a high-density line in visible (**a**). At stage B, a scalloped high-density line can be visualized (**b**). At stage C, two parallel, scalloped, high-density lines are visible, that are separated by some small, low-density areas (**c**). At stage D, two scalloped lines can be seen; however these lines cannot be visualized in the palatine bone, and the parasutural palatine bone density is higher when compared with the parasutural maxillary bone density (**d**). At stage E, the MPS cannot be visualized as the fusion has occurred, and the density of the bone is homogeneous (**e**).

First, we aimed to reach agreement on MPS maturation stages that can be used as ground truth. In order to do that, the raw CBCT images (without modification) were randomly ordered into a Microsoft PowerPoint presentation for analysis. In total, 9 experienced orthodontists with an average of 13.11 years of clinical experience were chosen to analyze the images. None of them participated in planning the study design. The raters were asked to determine the maturation stage of the MPS according to the method described previously by Angelieri et al. [8]. Briefly, MPS maturation can be divided into five maturation stages, stages A–E (Figure 1a–e), according to the number, shape, location and visibility of high-density lines representing the MPS on the CBCT images.

The responses were then recorded with a digital evaluation form using Google Forms. Along with the form, each examiner received a detailed description and exemplar CBCT images of MPS maturation stages as reference material during evaluation. At each CBCT, the reference material reappeared again on the Google Form along with the answer options. The examiners were then asked to evaluate the identical scans twice, at a two-week interval between the first and second rating. For the latter session, the examiners received identical instructions and materials as the initial survey, except the image order was randomly reorganized. According to their results, examiners with the highest strength of agreement were selected and the final stage agreement was based on their evaluation (see details in Section 2.4).

### 2.4. Statistical Analysis

First, Fleiss’ and Cohen’s Kappa coefficients were calculated to assess the inter- and intra-examiner reliability of the 9 examiners. Based on Kappa values [17], the interpretation of the results for the strength of agreement is shown in Table 2. For better visualization, the different levels of agreements were color coded. A Kappa value of 0 indicated no agreement (white), values ranging from 0 to 0.20 poor agreement (yellow), values from 0.21 to 0.40 fair (orange), from 0.41 to 0.60 moderate (blue), 0.61–0.80 good (purple) and between 0.81 and 1.00 very good agreement (green).

**Figure 2:**
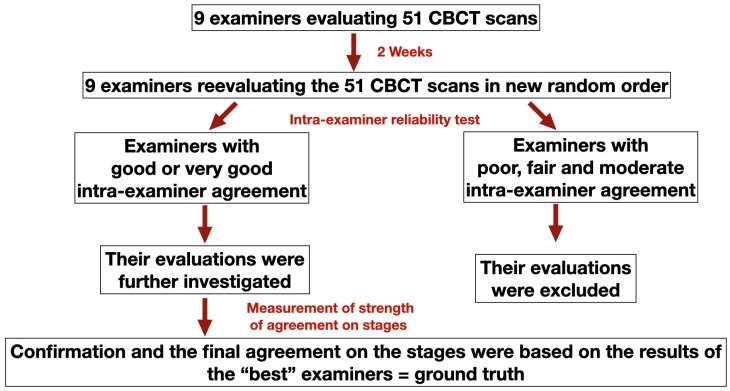
Flowchart of the decision-making protocol of the final agreement on the MPS staging.

Following the two-round evaluation, the final agreement and confirmation of the maturation stages were based on the following protocol: Results of those examiners whose intra-examiner reliability score was worse than good or very good were excluded from further evaluation. After that, the strength of agreement on each stage was measured for those examiners who showed either good or very good intra-examiner reliability and the staging results were accepted as ground truth. Figure 2 demonstrates the flow chart of the decision-making protocol of the final agreement on the MPS staging.

The confirmed stages were then used to examine differences in fractal dimension values using one way analysis of variance (ANOVA) with Tukey’s post hoc test. 

Following that, stages A, B, and C were separated from D and E for further evaluation, as this was considered as the boundary for the MPS maturation. Therefore, based on the empirical distribution function of each parameter, the degree of discrimination between the two groups (stage A, B, C vs. D, E) was obtained by using a receiver operating characteristic (ROC) curve with the AUC (area under the curve). The AUC ranges from 0.5 (random prediction) to 1 (perfect discrimination). The optimal cut-off point of the fractal dimension that optimizes the sum of sensitivity and specificity was then determined as well. All data are presented as mean ± standard deviation (SD) values. SPSS software (IBM Corp. Released 2021. IBM SPSS Statistics for Windows, Version 28.0. Armonk, NY, USA: IBM Corp) was used and *p* < 0.05 was considered statistically significant.

## 3. Results

### 3.1. Intra- and Inter-Examiner Reliability of MPS Maturation

The overall inter-examiner agreement for the first round of the test is summarized in Table 3. The overall agreement on the first test was found to be quite weak with a Kappa value of 0.268, which indicates a ‘fair’ agreement. Although there was a slight increase compared to the first test, the results of the second test displayed again fair agreement with a Kappa value of 0.349 (Table 3).

As for the intra-examiner reliability, the Cohen’s Kappa results are presented in Table 4 for each examiner. The results varied from poor to good in terms of Kappa scores. Two raters out of nine had poor, one examiner fair, two moderate and four good agreements. The Kappa value for the poorest reliability was found to be 0.126, while the result of the strongest agreement was good, with a Kappa score of 0.715 (Table 4).

### 3.2. Agreement on MPS Stages and Fractal Dimension

None of the examiners had very good intra-examiner reliability. Therefore, the final agreement on stages for each CBCT was based on the results of the second test of the four raters with good reliability. Then the strength of agreement on each stage was assessed between these four raters. Table 5 shows the results of the strength of agreement on each stage. 

The strength of agreement was good only for stage D and moderate for the rest of the stages. The stage D for these CBCTs was accepted and confirmed. However, in case of CBCTs where the stage agreement did not reach a good level, the final agreements on the MPS stages were based on the consensus of the authors being expert in this field. Namely, CBCTs were evaluated by two experts (authors of the paper) blinded for the results of the test and in case of disagreement a third expert was asked to reach the final agreement.

**Figure 3:**
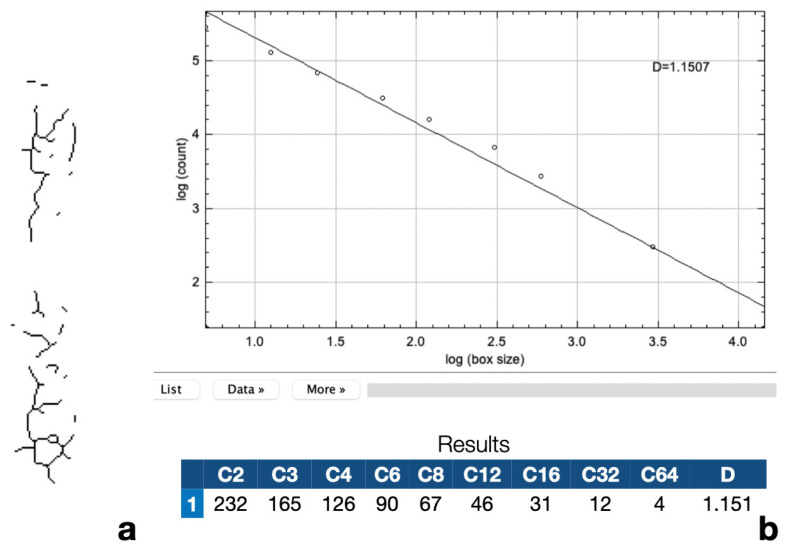
Representative picture of a skeletonized MPS (**a**) and the result of fractal analysis (**b**).

The confirmed stages were then paired with the fractal dimension values. Figure 3a,b demonstrates a representative picture of a skeletonized MPS and the result of fractal analysis.

All in all, from all the individuals being examined (as mentioned in Table 1), 2 subjects were stage A, 7 subjects belonged to Stage B, 23 to Stage C, 13 to Stage D and 6 to Stage E, and all showed statistically significant differences in fractal dimension values (Table 6).

**Figure 4:**
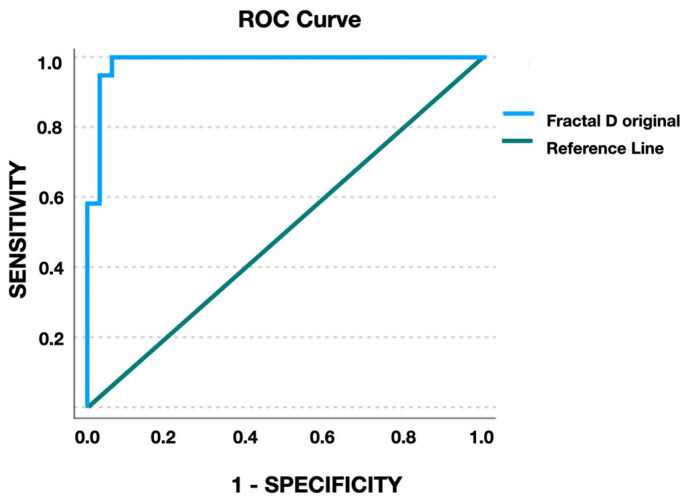
ROC curve of fractal dimension.

To test the maturation boundary between the maturation stages of A, B, C vs. D, E, the ROC curve (Figure 4) was developed. On the ROC curve, the sensitivity is plotted on the *Y*-axis, representing the true positive rate, while the 1-specificity is on the *X*-axis representing the false negative rate. By developing the curve, the cut-off point for fractal dimension—the value that maximizes the sensitivity and specificity—was found to be 1.073. The area under the curve (AUC) showed a high result, 0.985 (CI: 0.957–1.000, *p* < 0.0001), so this fractal dimension was found to be a statistically significant indicator.

At this cut-off point, the use of the fractal dimension value for predicting MPS maturation stage showed 100% sensitivity, 93.7% specificity, 9.5% false positive rate, 0% false negative rate, 91.15% positive predictive value, and 0% negative predicted value.

## 4. Discussion

Medical specialists have always sought more reliable diagnostic methods to conclude proper therapeutic decisions. For orthodontic problems, accurate diagnosis and proper treatment plans with correct timing greatly rely on the clinician’s ability to predict the patient’s future growth patterns. During orthodontic diagnosis, an individual analysis should be carried out to promote the best treatment option as the onset of treatment timing may be critical [18]. In the present study, we aimed to test the diagnostic applicability of the fractal analysis for quantitative analysis of MPS maturation. We found that the method itself has good sensitivity and specificity, which could make it a potential adjunct diagnostic approach for determining the maturation boundary of the MPS.

Transversal maxillary deficiencies are very common orthodontic anomalies [19], as their prevalence is approximately 10% in adults [20] and is often characterized by abnormal bucco-lingual relationship of the posterior teeth, such as unilateral or bilateral crossbite. Bad habits such as thumb sucking, use of pacifiers, mouth breathing, abnormal or atypical tongue function, swallowing, and imbalanced perioral muscles are among the most common etiologic factors that can contribute to the development of transversal orthodontic anomalies.

When treating transversal problems, the patient’s response to expansion (skeletal versus dental) is a significant factor that should be considered by every orthodontist. As far as transversal problems often present as a maxillary constriction, the orthodontic correction should aim for the maximum amount of skeletal expansion and a minimum amount of dentoalveolar expansion. Since 1860, when Angell first described the RME protocol, many other procedures, along with tooth-borne, hybrid and bone-borne devices, have been introduced to correct the constricted maxillary arch [21]. With expansion, not only the transversal problem can be solved, but smile aesthetics (buccal corridors), occlusal contacts, functions, and sagittal problems can be improved as well. Additionally, during expansion, crowding can be resolved due to the increase in the arch perimeter and patients with transversal deficiency might further benefit from the improvement in upper airway volume [4].

However, for the appropriate treatment choice of transversal discrepancies, the precise determination of MPS maturation is crucial. Namely, when the MPS and the adjacent articulations begin their fusion and interdigitations appear, it will become more rigid and will exhibit higher resistance to expansion forces [4,11,22]. Therefore, conventional RME treatment could result in failure and/or increased dental effects, such as buccal crown tipping, gingival recession, reduction of buccal bone thickness, alveolar bone dehiscence, fenestration, marginal bone loss, root resorption and pain, all of which are undesired side-effects of RME [23,24,25,26]. Moreover, it was reported that age alone cannot be a reliable indicator for predicting the fusion of MPS, as even in adults it was found non-matured [13,27]. To overcome limitations, an individual assessment of MPS maturation would be beneficial for the optimal choice of treatment.

For evaluation of skeletal maturation itself, several methods have been used, such as increase in statural height, hand–wrist radiographs, and cervical vertebral maturation using lateral cephalograms [12]. In 2013, a novel classification method for the determination of MPS maturation was developed by Angelieri et al. [8] to design the best treatment option and timing. Their method relies on the morphological characteristics of the MPS. However, Barbosa et al. concluded that it should be applied with caution for routine daily use as it was not reliable enough [7]. Furthermore, they recommended the need for an extensive training program for routine clinical application of the method [7]. Another systematic review that aimed to assess the methodological quality of all the articles about Angelieri’s method also highlighted caution when using Angelieri’s classification routinely [28].

Since 2013, several methods have been proposed for the evaluation of MPS maturation. However, most of them were proved to have limitations and none of them has been proved to be a valid for the determination of MPS maturation and prediction of the amount of achievable skeletal expansion [12,27]. For example, MPS density appeared to be a potential indicator, but problems with CBCT machine standardization seemed to be rather difficult [12]. In addition, Gao et al. reported a quantitative characteristics analysis for MPS maturation which uses a machine learning algorithm. This method seems to be promising; however, as suggested by the authors, the method needs further improvements, which can be achieved by implementing an increased sample size and enhanced sample representation [29]. In 2016, another method was published for potential use which utilized quantitative fractal analysis to determine the MPS maturation stage [13]. However, its validity and applicability have not been investigated. 

Fractal analysis is a mathematical method that can be used to describe and analyze structural patterns and complex shapes such as bone, and it has been used in all areas of science, including dentistry [30]. It has been shown to be able to evaluate the healing of endodontic lesions following root canal treatment [31], quantify trabecular changes after bone regeneration of the jaw [32], diagnose caries [33], and to evaluate the morphological pattern of jawbones and its possible change over time [34].

In this present study, we aimed to provide further evidence as to whether fractal analysis can be used to determine proper MPS stages. In order to do that, first we aimed to assess the MPS stages most precisely according to Angelieri’s method. Therefore, orthodontic specialists with an average experience of thirteen years were asked to evaluate fifty-one MPS CBCT images on two different occasions with randomized orders using Angelieri’s classification method. The results were then analyzed to assess inter- and intra-examiner reliability agreements. These findings were then reflected in Fleiss’ and Cohen’s Kappa statistic. According to the results, the overall inter-examiner agreement was ‘poor’ for both tests and the intra-examiner agreement also varied greatly ranging from ‘poor’ to ‘good’ strength in terms of agreements, indicating the low reproducibility of the Angelieri’s MPS staging method. This was in accordance with the results of others [7,13]. However, a reliable procedure should always result in similar outcomes regardless of the time and environment of the assessor [35]. Therefore, to minimize error, we excluded all the raters with low agreement and, based only on the results of the best examiners, the stages were then confirmed and used as ground truth. Then the stages were paired together with fractal dimension values and the maturation boundary was determined. According to a previous study carried out by Angelieri, the maturation stages were separated in two groups (A–C vs. D, E) to demonstrate the boundary of MPS maturation. We aimed to find the fractal dimension value that could represent that boundary by developing the ROC curve. As the AUC was found to be high, the cut-off point can be used to determine the maturation boundary of MPS. Our cut-off point was 1.073, slightly higher (0.0495) that what was found by Kwak et al. The difference could be explained by different ROI (region of interest) selection and image processing. The selection of the ROI should be performed carefully and precisely to avoid the inclusion of greater areas around the MPS. In our study, one examiner chose the ROI for all the CBCT images; however, differences in the image cropping could slightly influence the fractal dimension values as well. Another possible explanation could be the biological nature of the maturation process itself, as it does not occur from one stage to another suddenly but rather gradually, so the difference could be the result of the transition phase as well. One of the major disadvantages of this method is that there is a high demand for the clinicians to acquire competence in image processing. In addition, it has been suggested that the cost, resources, and knowledge required for fractal analysis might make this method impractical in a clinical practice [36]. Moreover, clinical failure of maxillary expansion could be a result of the resistance of not only the fused MPS but also other neighboring structures such as zygomatic buttress, piriform aperture, zygomaticotemporal, zygomaticomaxillary and pterygopalatine sutures. Accordingly, to make a proper diagnosis, the use of more than one method is recommended. 

In summary, having low reproducibility in Angelieri classification may propose limitations when developing optimal clinical decisions. Fractal analysis showed good diagnostic performance and seems to be a promising adjunct diagnostic method; therefore, along with the Angelieri classification method, it could display useful information for orthodontic diagnosis and treatment planning.

## 5. Conclusions

Our results suggest that fractal analysis is a potential and useful diagnostic tool to quantitatively evaluate the MPS as it showed good diagnostic performance. Therefore, this method might provide further objective and reliable parameters for clinicians on the decision for the most appropriate treatment of skeletal transversal maxillary deficiencies.

## Figures and Tables

**Table 1:** Age and gender distribution of the subjects.

	Men	Women
Number of subjects	19	32
Average age (years)	21.79 ± 6.6	23.1 ± 6.26
Age 9–15 (years)	3	3
Age 16–20 (years)	6	8
Age 21–25 (years)	6	10
Age 26–30 (years)	2	8
Age 31–40 (years)	2	3

**Table 2:** Interpretation of Fleiss’ and Cohen’s Kappa statistics.

Value of Kappa	Interpretation (Strength of Agreement)
0	No agreement
<0.20	Poor
0.21–0.40	Fair
0.41–0.60	Moderate
0.61–0.80	Good
0.81–1.00	Very good

**Table 3:** Fleiss’ Kappa values for inter-examiner overall agreement of Test #1 and #2. The color coding of table was used for better visualization of the results.

	Strength of Agreement (Kappa Value)
Test # 1	0.268 (Fair)
Test # 2	0.349 (Fair)

**Table 4:** Cohen’s Kappa values for intra-examiner reliability. The color coding of table was used for better visualization of the results.

Examiner	Strength of Agreement (Kappa Value)
1	0.625 (Good)
2	0.631 (Good)
3	0.412 (Moderate)
4	0.711 (Good)
5	0.306 (Fair)
6	0.138 (Poor)
7	0.381 (Moderate)
8	0.126 (Poor)
9	0.715 (Good)

**Table 5:** Strength of agreement on each stage between the four examiners with good intra-examiner reliability measured by Fleiss’ Kappa. The color coding of table was used for better visualization of the results.

MPS Stage	Strength of Agreement on Individual Stages
Stage A	0.345
Stage B	0.460
Stage C	0.474
Stage E	0.438
Stage D	0.690

**Table 6:** Fractal dimension values for each MPS (mean ± SD).

MPS Stage	Fractal Dimension (mean ± SD)	95% CI Lower Bound	95% CI Upper Bound
Stage A	1.267 ± 0.015	1.133	1.399
Stage B	1.197 ± 0.039	1.161	1.233
Stage C	1.095 ± 0.29	1.082	1.108
Stage E	1.017 ± 0.019	1.001	1.029
Stage D	0.973 ± 0.034	0.938	1.008

## Data Availability

Data sharing not applicable.
